# Genetic Regulation of Monocyte MicroRNAs and Their Implication in Musculoskeletal Diseases: A Cross-Ancestry Expression Quantitative Trait Loci and Imputation Study

**DOI:** 10.3390/ijms27062818

**Published:** 2026-03-20

**Authors:** Yong Liu, Kuan-Jui Su, Yun Gong, Bo Tian, Anqi Liu, Zhe Luo, Qing Tian, Chuan Qiu, Hui Shen, Hong-Mei Xiao, Hong-Wen Deng

**Affiliations:** 1 Center for System Biology, Data Sciences, and Reproductive Health, School of Basic Medical Science, Central South University, Changsha 410031, China; liuyong20@csu.edu.cn (Y.L.);; 2 Tulane Center of Biomedical Informatics and Genomics, Deming Department of Medicine, School of Medicine, Tulane University, New Orleans, LA 70112, USA; 3 Institute of Reproductive & Stem Cell Engineering, School of Basic Medical Science, Central South University, Changsha 410000, China

**Keywords:** microRNA, eQTL, monocyte, imputation model, musculoskeletal diseases

## Abstract

This study investigated the genetic regulation of microRNA (miRNA) expression in monocytes and its potential role in musculoskeletal diseases. We mapped expression quantitative trait loci (eQTLs) for miRNAs using data from 281 Caucasian (CAU) and 170 African American (AA) individuals, constructed ancestry-specific models to impute miRNA expression from genotype data, and applied these models to test associations with osteoporosis and sarcopenia. Analysis identified 468 and 2653 independent eQTLs for 61 miRNAs in CAU and 25 in AA, respectively, the majority of which were ancestry-specific. Association analyses identified 22 and 26 miRNAs associated with osteoporosis and sarcopenia, respectively, in the CAU population; corresponding findings in the African American population were 26 and 14 miRNAs. Analysis of their target genes revealed 1238 and 741 genes that were nominally associated with osteoporosis and sarcopenia in CAU; with 524 genes associated with osteoporosis and 891 associated with sarcopenia in AA. Functional enrichment analysis indicated that the target genes of the identified miRNAs are involved in disease-relevant biological processes—cell migration and motility in osteoporosis, and immune/cytokine responses in sarcopenia. This work provides insights into the genetic architecture of miRNA expression and implicates monocyte miRNAs in musculoskeletal diseases, underscoring the importance of including diverse ancestral backgrounds in genomic studies.

## 1. Introduction

Genome-wide association studies (GWASs) have cumulatively identified numerous loci containing common genetic variants associated with complex human diseases [1,2]. Since the majority of these variants reside in non-coding regions [3,4], research has increasingly focused on understanding how gene expression regulation mediates genetic influences on complex traits. A comprehensive understanding of disease mechanisms will ultimately require the integration of multi-omics data across diverse molecular [4].

MicroRNAs (miRNAs) are small non-coding RNAs of approximately 22 nucleotides that post-transcriptionally regulate gene expression through complementary base-pairing with target mRNAs [5]. Following transcription and stepwise processing into mature forms, miRNAs are loaded into the RNA-induced silencing complex alongside Argonaute proteins [5,6], leading to translational repression or degradation of target transcripts [6]. By regulating critical processes including development, apoptosis, and metabolism, miRNAs play a key role in cellular fine-tuning [7]. Consequently, their dysregulation is implicated in a broad spectrum of diseases, including cancers, neurodegenerative disorders, and cardiovascular conditions [8,9,10]. As such, they represent promising candidates for both biomarkers and therapeutic targets [11].

Monocytes are pivotal circulating immune cells that orchestrate innate immune defense through pathogen clearance, intercellular signaling, and tissue repair—processes essential for infection control and inflammatory homeostasis [12]. Beyond their classical immunological roles, monocytes have emerged as key mediators in the pathogenesis of musculoskeletal diseases, such as osteoporosis and sarcopenia. These diseases are common complex diseases that are biologically interconnected through shared inflammatory and metabolic pathways [13]. Circulating monocytes act as a key role in this shared pathophysiology, serving as an important source of inflammatory cytokines that can affect the function of bone and muscle [14]. In osteoporosis, monocyte-derived osteoclasts directly execute bone resorption, and monocyte-secreted inflammatory cytokines disrupt the balance of bone remodeling [15]. Similarly, in sarcopenia, monocytes contribute to chronic low-grade muscle inflammation, accelerating proteolysis and impairing regenerative capacity [16]. Given the key role of monocytes in these interconnected diseases, alterations in their miRNA expression profiles can modulate target gene networks, thereby influencing key biological processes such as inflammatory signaling, energy homeostasis, bone remodeling, and muscle maintenance—thereby driving the pathogenesis of osteoporosis and sarcopenia [17].

Genetic variation contributes to miRNA dysregulation in psychiatric disorders primarily through mechanisms such as miRNA expression quantitative trait loci (miR-eQTLs)—genomic regions associated with individual differences in miRNA expression levels. These loci help explain how genetic variants influence miRNA abundance, which may in turn affect susceptibility to complex diseases [18]. Additionally, miRNA-related single nucleotide polymorphisms (SNPs), occurring within miRNA genes or their target sites, can disrupt miRNA biogenesis or target recognition, thereby altering miRNA–mRNA interactions and contributing to disease pathogenesis [19]. Despite the well-established role of mRNA eQTLs, which have been extensively mapped across diverse tissues and cell types, investigations of miR-eQTLs remain limited in the diversity of cellular contexts and the scale of cohorts. This gap constrains a systematic understanding of the genetic architecture of miRNA regulation and its contribution to complex traits.

Conventional miRNA profiling methods, including microarrays and next-generation sequencing, are constrained by inherent technical limitations. These comprise biases introduced during library construction—such as nucleotide preference of T4 RNA ligase and inefficient adapter ligation—GC-content heterogeneity leading to amplification bias, and significant challenges in the accurate detection of low-abundance miRNAs, particularly in samples with limited input RNA or high levels of impurities [20,21]. These issues contribute to elevated experimental costs and constrained sample size. In contrast, genomic datasets from large-scale biobanks offer exceptional scalability, such as the UK Biobank (UKBB), with over 500,000 whole genomes and aggregate sample sizes exceeding 15 million [22]. Recently, tools such as FUSION and PrediXcan have demonstrated success in constructing genetic prediction models for molecular traits such as mRNAs expression across diverse tissues and have been widely adopted in transcriptome-wide association studies (TWASs) [23,24]. However, such approaches remain notably underexplored in the context of miRNA research. By constructing miRNA expression prediction models from reference datasets that contain both genomic and miRNA sequencing profiles, we can then apply these models to much larger genetic cohorts which have genotype data but lack direct miRNA measurements. This framework enables computationally efficient, high-throughput miRNA-disease association studies at an unprecedented scale.

**Figure 1:**
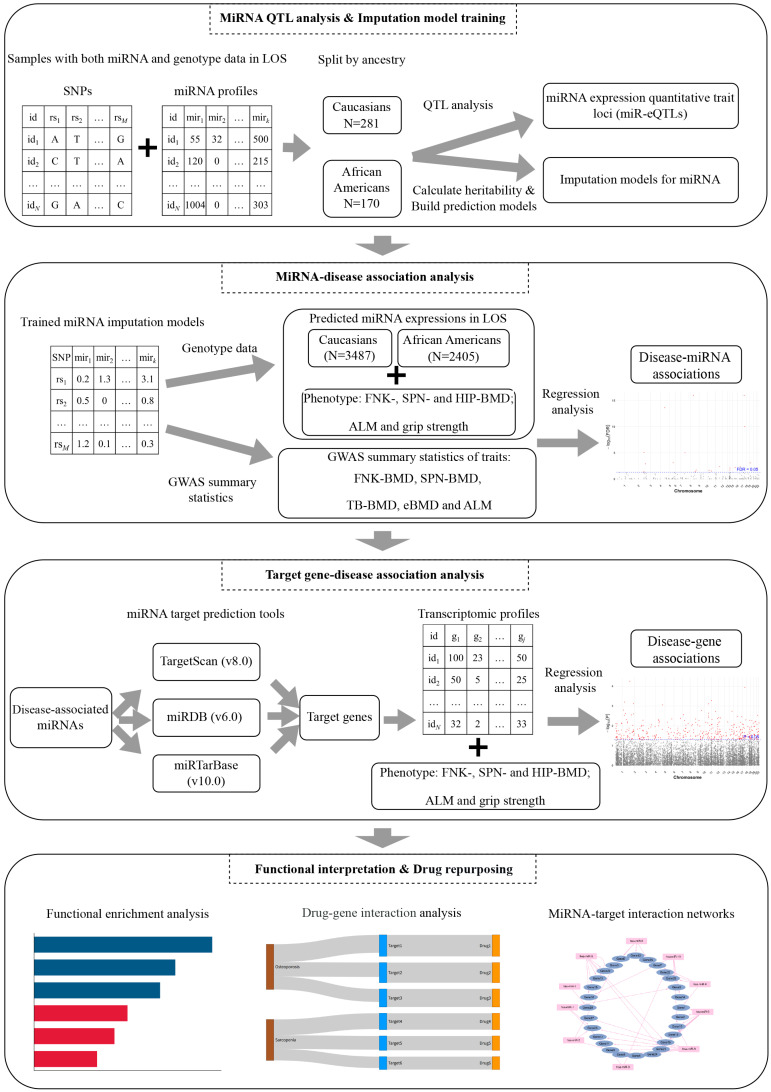
The workflow of our study. BMD refers to bone mineral density; ALM refers to appendicular lean mass; FNK refers to femoral neck; SPN refers to spine; eBMD refers to heel estimated BMD; TB-BMD refers to total body BMD.

In this study, we aimed to map miR-eQTLs in monocytes, construct imputation models to predict miRNA expression from genomic data, and leverage these models to perform association analyses with osteoporosis and sarcopenia. We first performed a QTL analysis to identify cis-regulatory variants influencing miRNA expression in both Caucasian (CAU) and African American (AA) populations from the Louisiana Osteoporosis Study (LOS) [25]. Then, we constructed ancestry-specific models to genetically predict miRNA expression levels for each population. These models were subsequently applied to internal genomic resources as well as external summary-level datasets to investigate the role of miRNAs in the etiology of osteoporosis and sarcopenia. Next, we leveraged public databases and online tools to identify the target genes of the disease-associated miRNAs. Further, we performed association analysis between these targets and disease traits using mRNA transcriptome data from the corresponding LOS populations to provide additional functional support. The overall study workflow is summarized in Figure 1. Our study provides comprehensive insights into the genetic architecture of miRNA regulation in monocytes and underscores its implications for musculoskeletal diseases. We create several data resources, comprising miRNA eQTLs and imputation models, will facilitate future investigations into miRNA-disease associations and exploration of the drug repurposing potential of miRNA-mediated pathways.

## 2. Results

### 2.1. Identification of Cis-miR-eQTLs Across Two Populations

**Figure 2:**
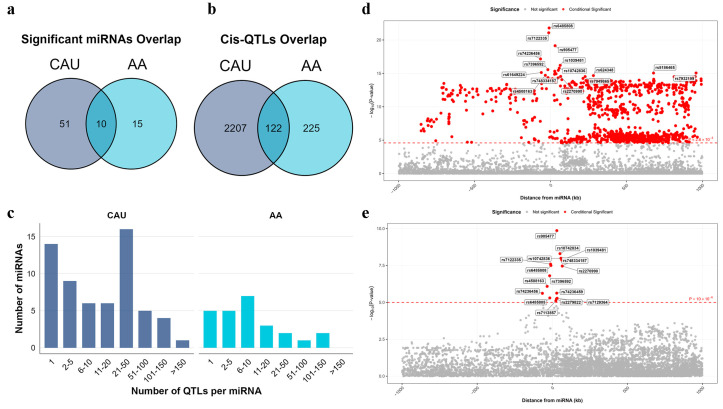
Results of miRNA QTL analyses. (**a**) Overlap of miRNAs with at least one significant cis-QTL between AA and CAU sub-population. (**b**) Overlap of significant cis-QTLs between AA and CAU sub-population. (**c**) Summary of number of cis-QTLs per miRNA. (**d**) Manhattan plot for cis-QTL results of has-mir-3161 in CAU sub-population (n = 281). (**e**) Manhattan plot for cis-QTL results of has-mir-3161 in AA sub-population (n = 170). Cis-miR-eQTL mapping was performed using QTLtools (v1.3.1). Association testing was conducted using the adaptive permutation procedure implemented in QTLtools to correct for multiple testing. For miRNAs with significant cis-eQTLs, independent regulatory signals among the proximal SNPs were further identified using the conditional analysis approach in QTLtools. Significant miRNA-eQTLs were defined as those passing a false discovery rate (FDR) threshold of <0.05. The red dotted line represents the significance threshold. CAU refers to Caucasian; AA refers to African American.

We identified 136 and 92 miRNAs with at least one significant (False discovery rate [FDR] < 0.05) cis-miR-eQTL near the pre-miRNA in CAU and AA sub-population, respectively (Tables S1 and S2). Using stepwise regression, we identified multiple conditional independent cis-SNPs for 61 (45%) of these miRNAs in the CAU population and 25 (27%) in the AA population, collectively defining 2653 (CAU) and 468 (AA) independent cis-miR-eQTLs, respectively (Tables S3 and S4). Among these, 10 miRNAs and 122 eQTLs were common to both populations (Figure 2a,b). The majority of miRNAs were associated with fewer than 100 independent cis-miR-eQTLs in both populations (Figure 2c). The miRNA hsa-mir-3161 (located on chromosome 11) exhibited the largest number of cis-miR-eQTLs in our analysis, with 1079 and 15 independent eQTLs identified in CAU and AA populations, respectively. Despite this substantial disparity in the number of significant associations, both populations shared several top-associated SNPs, such as rs905477, rs6485808, and rs7122335, suggesting the presence of a shared genetic regulatory mechanism (Figure 2d,e). The difference in the number of detected eQTLs likely reflects the larger sample size available in the CAU cohort, which provides greater statistical power to identify associations with moderate effects. Additionally, the ancestry-specific differences in linkage disequilibrium (LD) patterns and allele frequencies between the CAU and AA populations may also contribute to the observed disparity in the identified eQTLs.

### 2.2. Cis-Heritability of MiRNAs and Imputation Model Construction

**Figure 3:**
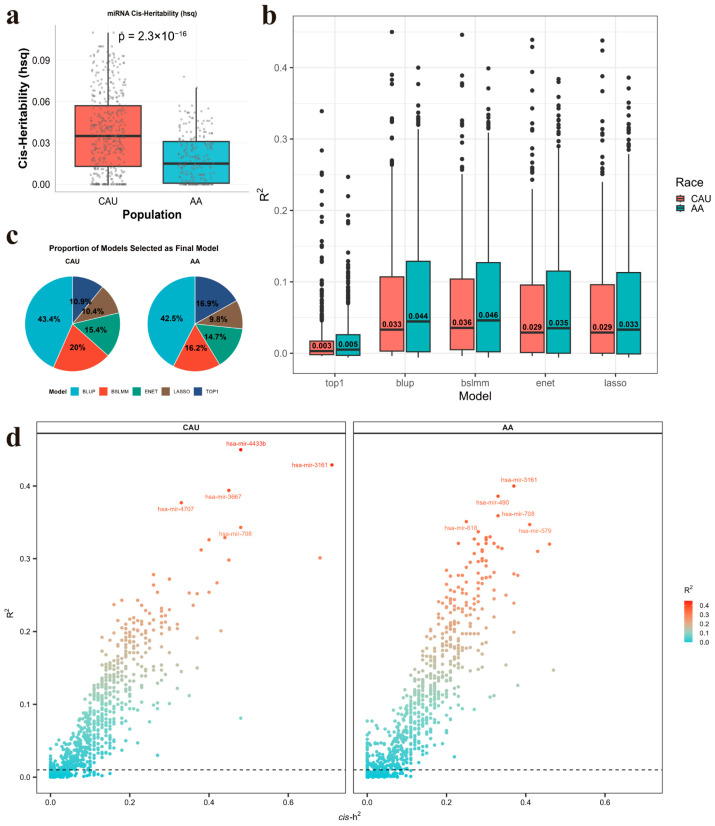
Performance of miRNA imputation models. (**a**) Comparison of miRNA cis-heritability between AA and CAU sub-population. (**b**) Average predictive model performance (R2) for each prediction model. (**c**) The proportion of each model selected as the best predictive model. (**d**) The relationship between cis-heritability and predictive model performance (R2). CAU refers to Caucasian; AA refers to African American.

We observed that the median cis-heritability (cis-h^2^) of miRNAs was significantly lower in AA (0.015) than in CAU (0.035; Wilcoxon *p* < 0.001; Figure 3a). We also observed consistent patterns across both populations: the TOP1 model demonstrated substantially lower mean R^2^ compared to other methods, reflecting the polygenic architecture underlying miRNA expression regulation (Figure 3b). Among the five models evaluated, BLUP emerged as the most robust and effective predictor of miRNA expression across ancestries (Figure 3c). Furthermore, we observed that miRNAs with higher cis-h^2^, often supported by a larger number of informative cis SNPs in both ancestry groups, exhibited better imputation performance (Figure 3d). For downstream association analyses, we retained miRNAs with a cross-validated R^2^ ≥ 0.01 and *p* < 0.05, which resulted in 249 miRNAs in CAU and 217 in AA.

### 2.3. Associations of Imputed MiRNAs with Musculoskeletal Diseases

**Figure 4:**
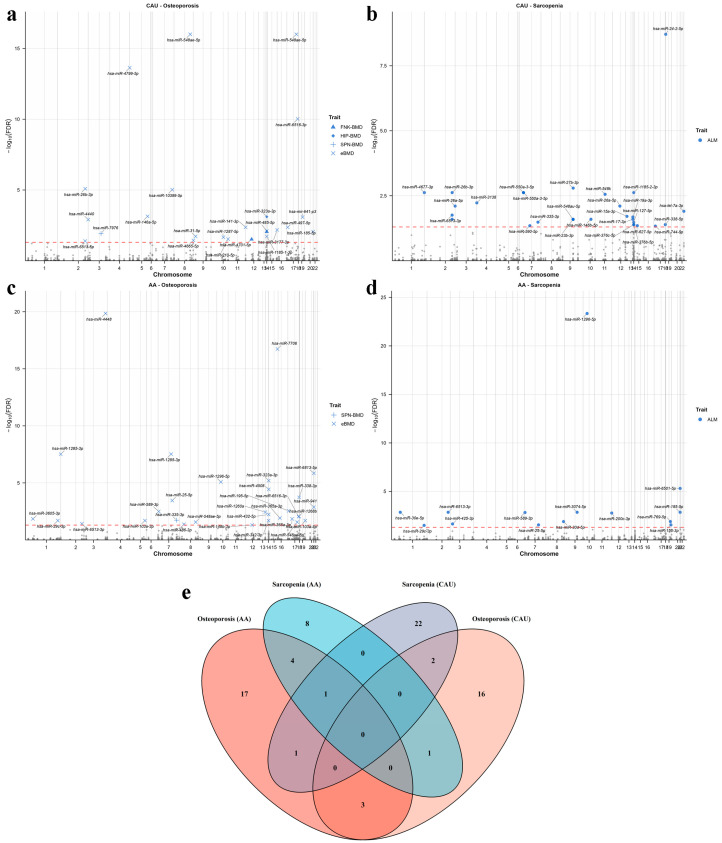
Results of association analysis between predicted miRNAs and musculoskeletal diseases. (**a**) Manhattan plot for association analysis between miRNAs and osteoporosis traits with CAU models. (**b**) Manhattan plot for association analysis between miRNAs and sarcopenia traits with CAU models. (**c**) Manhattan plot for association analysis between miRNAs and osteoporosis traits with AA models. (**d**) Manhattan plot for association analysis between miRNAs and sarcopenia traits with AA models. (**e**) Venn diagram of the intersection of disease-associated miRNAs between two ancestral subgroups. CAU refers to Caucasian; AA refers to African American. BMD refers to bone mineral density; ALM refers to appendicular lean mass; FNK refers to femoral neck; SPN refers to spine; eBMD refers to heel estimated BMD; In the Manhattan plot, significant miRNAs are labeled using different shapes to denote which trait they are associated with. The red dotted line represents the significance threshold.

We applied our trained miRNA imputation models to association analyses using both individual-level and GWAS summary-level data. MiRNAs significantly associated (FDR < 0.05) with bone mineral density (BMD) at femoral neck (FNK-BMD), spine (SPN-BMD), hip (HIP-BMD), total body BMD (TB-BMD) or BMD estimated from quantitative heel ultrasounds (eBMD) were defined as osteoporosis-related, while those significantly associated (FDR < 0.05) with appendicular lean mass (ALM) or grip strength were defined as sarcopenia-related. The association results for each trait are presented in Table 1 and Figure 4a–d. With CAU models, we identified 22 osteoporosis-related miRNAs and 26 sarcopenia-related miRNAs, respectively. With AA models, we identified 26 osteoporosis-related miRNAs and 14 sarcopenia-related miRNAs, respectively. The details of the significant association results are listed in Table S5.

Overall, we prioritized 75 unique miRNAs (45 osteoporosis-related and 39 sarcopenia-related) for downstream analysis. Among these, nine miRNAs showed significant associations with both conditions (Figure 4e): hsa-miR-335-3p, hsa-miR-589-3p, hsa-miR-6513-3p, hsa-miR-1296-5p, hsa-miR-25-5p, hsa-miR-29c-3p, hsa-miR-6513-5p, hsa-miR-185-5p, and hsa-miR-26b-3p. These miRNAs may exert pleiotropic effects on both osteoporosis and sarcopenia, and thus represent promising common therapeutic targets for both conditions.

### 2.4. Target Genes of Disease-Associated MiRNAs

Potential target genes of the 75 significant miRNAs were predicted using TargetScan (v8.0) [26], miRDB (v6.0) [27], and miRTarBase (Release 10.0) [28], yielding 13,408 unique targets. Given that multiple distinct miRNAs can converge to regulate a single gene target, we sought to identify genes at the highest risk of miRNA-mediated perturbation. We systematically ranked all putative target genes based on the number of trait-associated miRNAs targeting them, hypothesizing that genes with the highest targeting frequency are most susceptible to dysregulation and thus central to disease etiology.

**Figure 5:**
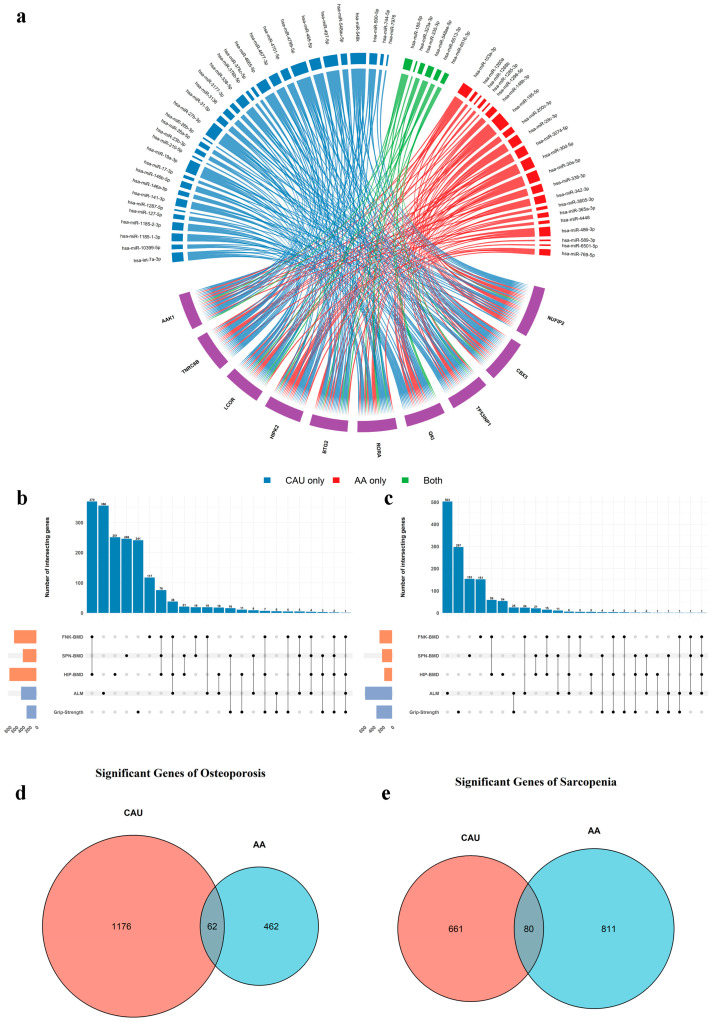
Results of association analysis between target genes and musculoskeletal diseases. (**a**) Circos-plot representing the 10 most targeted miRNA and the miRNAs that target them. The purple elements represent the target genes of miRNAs. (**b**) Upset plot displaying the count of target genes significantly associated with each trait and their overlaps in the CAU subgroup. (**c**) Upset plot displaying the count of target genes significantly associated with each trait and their overlaps in the AA subgroup. FNK-BMD, SPN-BMD, and HIP-BMD are traits related to osteoporosis, while ALM and Grip-Strength are traits related to sarcopenia. (**d**) Venn diagram showing the overlap of osteoporosis-associated genes between ancestry subgroups. (**e**) Venn diagram showing the overlap of sarcopenia-associated genes between ancestry subgroups. CAU refers to Caucasian; AA refers to African American. BMD refers to bone mineral density; ALM refers to appendicular lean mass; FNK refers to femoral neck; SPN refers to spine.

The top 10 genes ranked by miRNA targeting frequency are presented in Figure 5a. As expected, these genes are primarily involved in transcriptional/post-transcriptional regulation and the maintenance of cellular signaling and homeostasis. For instance, Quaking (*QKI*), an RNA-binding protein targeted by 25 trait-associated miRNAs, is a key regulator of pre-mRNA alternative splicing with diverse biological roles, including mRNA regulation, tumor suppression, and anti-inflammatory activity. Previous studies have linked *QKI* deficiency to osteoporosis through promotion of receptor activator of NF-κB ligand (RANKL)-induced osteoclastogenesis and disruption of bone metabolism [29]. *QKI* also influences striated muscle pathology via alternative splicing and has been implicated in mental and cardiac disorders [30,31,32]. Collectively, this evidence underscores the involvement of *QKI* in a broad spectrum of physiological and disease processes, highlighting its potential as a therapeutic target for treating multiple comorbidities simultaneously.

### 2.5. Association Between Target Gene Expression and Musculoskeletal Diseases

We examined associations between target gene expression levels and each trait across ancestry-specific subgroups. The number of genes significantly associated (*p* < 0.05) with each trait is summarized in Figure 5b,c. In the CAU subgroup, 1238 and 741 genes were nominally associated with osteoporosis and sarcopenia, respectively. In the AA subgroup, 524 and 891 genes showed nominal association with osteoporosis and sarcopenia, respectively. The details of the significant association results in each ancestry subgroup are listed in Tables S6 and S7. Among these, we identified 62 osteoporosis-related and 80 sarcopenia-related genes that were shared across ancestry groups (Figure 5d,e). These overlapping gene sets were prioritized for further functional annotation, as they are more likely to represent core, conserved disease mechanisms rather than population-specific regulatory effects.

### 2.6. Functional Enrichment of Target Genes

**Figure 6:**
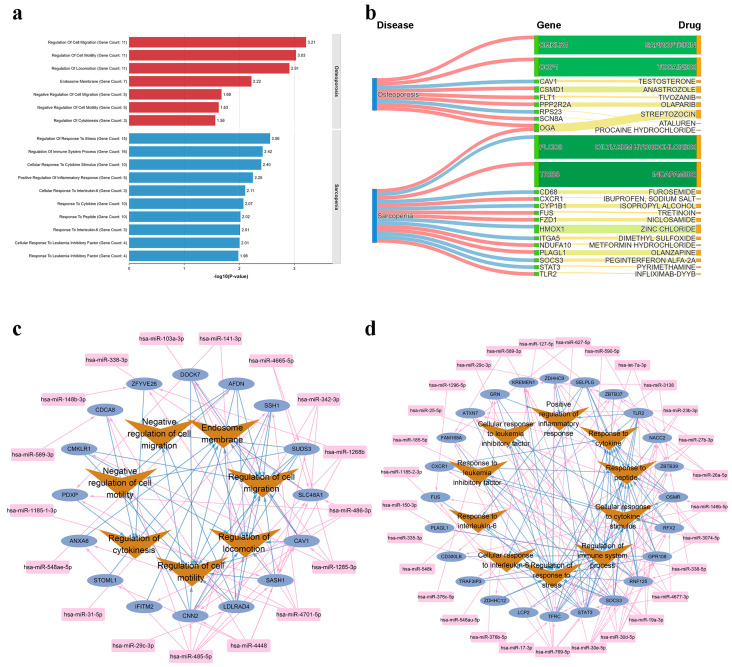
Functional interpretation of disease-associated genes. (**a**) Enriched GO terms for osteoporosis-related genes (7 significant terms) and top 10 most significantly enriched GO terms for sarcopenia-related genes. (**b**) Disease-gene-drug interaction network (only the drug with the highest interaction score is shown per gene). (**c**) MiRNA–target gene–functional module network for osteoporosis (based on the 7 significant GO terms). (**d**) MiRNA–target gene–functional module network for sarcopenia (based on the top 10 GO terms).

To elucidate the biological functions underlying the diseases, we performed Gene Ontology (GO) [33] enrichment analysis on the significant target genes. A total of 7 and 16 functional terms were significantly enriched for osteoporosis and sarcopenia, respectively (Table S8). The significantly enriched terms for each disease (Top 10 for sarcopenia) are displayed in Figure 6a. For osteoporosis, the enriched terms were predominantly related to the regulation of cell motility and migration, such as “Regulation of cell migration” (*p* = 6.14 × 10^−4^), “Regulation of cell motility” (*p* = 9.35 × 10^−4^), and “Regulation of locomotion” (*p* = 1.23 × 10^−3^). These findings suggest that the identified miRNAs may modulate monocyte migration and recruitment, a process essential for osteoclast precursor homing and subsequent bone resorption in osteoporosis pathogenesis [34]. For sarcopenia, the significantly enriched terms were largely associated with immune and inflammatory responses, as well as cytokine signaling. Notable terms included “Regulation of response to stress” (*p* = 2.76 × 10^−3^), “Regulation of immune system process” (*p* = 3.76 × 10^−3^), “Cellular response to cytokine stimulus” (*p* = 3.98 × 10^−3^), “Positive regulation of inflammatory response” (*p* = 5.65 × 10^−3^), and “Cellular response to interleukin-6” (*p* = 7.80 × 10^−3^). These results point to a role of miRNA-regulated genes in modulating inflammation and immune homeostasis, processes increasingly recognized as key contributors to muscle wasting in sarcopenia [35,36].

### 2.7. Disease-Gene-Drug Network

To investigate the therapeutic potential of miRNA-targeted genes, we queried the Drug-Gene Interaction Database (DGIdb) [37] to identify approved drugs associated with each candidate gene. We identified 96 drugs targeting 8 osteoporosis-related genes, and 47 drugs targeting 15 sarcopenia-related genes. For each gene, only the drug with the highest interaction score was retained to construct a disease–gene–drug network (Figure 6b). O-GlcNAcase (*OGA*) was associated with both osteoporosis and sarcopenia, and its related drug—Streptozotocin—was clinically used in the treatment of certain pancreatic islet cell cancers and experimentally employed to induce animal models of hyperglycemia, type 1 diabetes, or Alzheimer’s disease [38,39]. These findings indicate the possible utility of these drug–gene pairs for therapeutic repurposing. However, further functional and mechanistic studies are required to clarify their precise roles in disease pathophysiology, including the direction and magnitude of their effects.

### 2.8. MiRNA-Target-Function Network

To elucidate the regulatory relationships among miRNAs, target genes, and biological functions, we constructed miRNA-target gene-functional module networks for osteoporosis and sarcopenia based on the enriched GO terms (Top 10 for sarcopenia) along with their associated genes and miRNAs, and visualized the networks using Cytoscape (v3.10.4) [40]. As shown in Figure 6c,d, the osteoporosis network comprised 16 genes and 17 miRNAs, while the sarcopenia network contained 24 genes and 29 miRNAs. The observed pattern—where a single miRNA frequently targets multiple genes—highlights the potential of miRNAs as attractive candidates for intervening in complex, polygenic diseases such as osteoporosis and sarcopenia. By simultaneously modulating several genes within relevant pathways, miRNAs may offer more comprehensive and synergistic therapeutic effects compared to single-target agents.

## 3. Discussion

In this study, we performed a comprehensive analysis of the genetic regulation of miRNAs within the LOS cohort, stratified by CAU and AA ancestry. We developed the ancestry-specific miRNA expression prediction models for monocytes and provide these as a publicly available resource to facilitate future miRNA-disease association studies. By integrating in-house genotyping data with large-scale GWAS summary statistics for osteoporosis and sarcopenia-related traits, we illustrate how these models facilitate the identification of disease-related miRNAs and their target genes.

To our knowledge, this represents the first comprehensive miRNA eQTL analysis and expression prediction model construction in monocytes. In contrast to previous efforts using mixed cell types such as whole blood, our monocyte-focused approach offers a more precise cellular context, thereby improving functional interpretability and reducing confounding due to cellular heterogeneity. This work provides a milestone in our understanding of the genetic regulation of miRNAs and establishes a foundational, cell-type-specific resource, comprising eQTL maps and prediction models. This resource provides a critical path for future studies to dissect the role of miRNA in monocyte function and its contribution to monocyte-related diseases such as musculoskeletal or inflammatory disorders.

We observed substantial differences in cis-miR-eQTL profiles between the CAU and AA populations, with a high proportion of ancestry-specific signals. Among the 837 miRNAs analyzed, we identified independent cis-miR-eQTLs for 61 miRNAs in CAU and 25 in AA, corresponding to 468 and 2653 independent eQTLs, respectively. Notably, only 10 miRNAs and 122 eQTLs were shared across both groups, highlighting considerable population-specificity in miRNA genetic regulation. This disparity likely reflects underlying differences in genetic architecture between ancestries. Specifically, variations in allele frequencies and LD patterns between CAU and AA populations can substantially influence eQTL detection [41,42]. For instance, causal variants that are common in one population may be rare in another, reducing statistical power for detection even when sample sizes are comparable. Additionally, ancestry-specific LD structures can affect the fine-mapping resolution and the number of independently associated signals identified. Furthermore, part of this discrepancy may be attributable to differences in sample size between the cohorts. For instance, while hsa-mir-3161 shared several top-associated independent cis-miR-eQTLs in both populations, the number of statistically significant loci detected was substantially greater in the CAU group. This discrepancy is likely attributable primarily to the larger sample size and thus greater statistical power in the CAU population, despite the potentially higher discovery potential inherent in the more diverse genetic architecture of the AA population [43]. These observations underscore two critical points for future research: first, the importance of including ancestrally diverse cohorts to capture population-specific genetic architecture; and second, the necessity of adequate sample sizes to ensure robust detection of genetic regulators. Together, these considerations are crucial for generating molecular genetic insights that are both statistically reliable and broadly representative across diverse populations.

By integrating our miRNA imputation models with both individual-level genotyping data from the LOS cohort and publicly available GWAS summary statistics, we increased our total sample size, enabling us to identify 45 miRNAs significantly associated with osteoporosis and 39 significantly associated with sarcopenia. This approach confirmed a complex interplay between disease-associated genetic variation and miRNA expression. By identifying these genetically influenced miRNAs, we uncovered new candidate genes that may contribute to musculoskeletal disorders like osteoporosis and sarcopenia. This work also helps clarify the molecular pathways—both shared and distinct—that underlie these conditions. The rationale is that these miRNAs are possibly acting as a molecular bridge, translating genetic differences into disease risk by regulating the expression of target genes. These findings significantly advance our understanding of the genetic regulation of miRNAs in a cell-type-specific context and their potential roles in the pathophysiology of musculoskeletal diseases.

The ability of miRNA to regulate hundreds of mRNAs simultaneously, offer a unique therapeutic avenue for modulating multiple components of disease-associated gene networks [44]. We further identified the target genes of the detected disease-related miRNAs and prioritized genes significantly associated with osteoporosis or sarcopenia using transcriptomic data. Subsequent functional exploration, including GO enrichment analysis and the construction of miRNA-gene-functional module regulatory networks, revealed distinct biological processes associated with musculoskeletal disorders. For osteoporosis, the prioritized genes were predominantly involved in the regulation of cell motility and migration. Regulation of cell motility and migration mediates the recruitment of circulating monocytes—the precursors of osteoclasts—to the bone surface, an essential step in osteoclastgenesis and bone resorption [34]. Indeed, monocyte migration and homing to the bone microenvironment represent critical early stages in the development of osteoporosis, regulated by multiple signaling molecules [45]. Dysregulation of these migratory processes can directly influence osteoclast precursor recruitment and subsequent bone loss. For sarcopenia, the enriched terms were largely associated with immune and inflammatory responses, such as “cellular response to cytokine stimulus,” “response to interleukin-6,” and “regulation of inflammatory response”. These findings align with growing evidence that chronic inflammation plays a central role in the pathogenesis of age-related muscle wasting [35,36,46]. In particular, the responsiveness of monocytes to skeletal muscle-derived interleukin-6 signaling plays a critical role in mediating communication between the skeletal muscle and the immune system [47,48].

By targeting multiple genes within these functional modules, miRNA-based interventions offer the potential to modulate entire disease-relevant networks rather than individual effectors. This network-level regulatory capacity is particularly advantageous for complex, multifactorial diseases such as osteoporosis and sarcopenia, where multiple interconnected pathways contribute to disease progression. While precisely delineating the effect size and direction of these gene expression changes across different systems remains a challenge, such network-level intervention offers a more comprehensive and potentially more effective treatment strategy, overcoming the limitations of single-target therapies [8].

A major strength of our study is that the generated miRNA eQTL resources and imputation models can be flexibly utilized in the study of other diseases or phenotypes in various conditions. For example, in cohorts with only genotype data, miRNA expression profiles can be imputed to perform miRNA–trait association analyses. Alternatively, in the absence of individual-level genotype data, miRNA–disease associations can be examined using TWAS/FUSION approaches by integrating publicly available GWAS summary statistics [23]. Furthermore, Mendelian randomization can be employed to infer potential causal relationships between miRNAs and disease of interest [49]. Finally, these resources enable the exploration of drug-repurposing opportunities by targeting miRNA-mediated regulatory pathways.

This study has several limitations. First, our eQTL analysis and prediction model training were conducted exclusively in male samples, whereas the disease association analyses included both male and female participants. Future studies incorporating both sexes are needed to validate the identified disease-associated miRNAs. Second, our analysis was restricted to known human miRNAs from existing databases; subsequent research should be expanded to include newly discovered miRNAs. Third, we did not systematically explore trans-eQTLs affecting miRNA expression. Beyond cis-regions, genetic variants in broader genomic regions may influence miRNA expression, but identifying such trans-associations and incorporating them into prediction models would require even larger sample sizes to mitigate false positives and model overfitting. may also be highlighted. Fourth, the majority of GWAS summary statistics are derived from European-ancestry populations. The use of European-based GWAS data with African-ancestry LD references may lead to reduced power or spurious associations due to differences in linkage disequilibrium patterns and allele frequencies between ancestries. Future TWASs will benefit from the growing availability of large-scale, non-European GWAS cohorts, which will enable more accurate ancestry-matched analyses and improve the generalizability of findings across populations.

## 4. Materials and Methods

### 4.1. Study Population

This study utilized individual-level data from the LOS, an ongoing cross-sectional investigation initiated in 2011 that has enrolled over 17,000 participants to date to examine genetic and non-genetic determinants of osteoporosis and related complex diseases [25]. At current, whole-genome sequencing (WGS) was performed on blood samples from 7221 LOS participants. From this cohort, a male subset (n = 956) subsequently underwent whole-transcriptome sequencing (WTS), which included quantitative mRNA and miRNA expression profiling. To reduce cellular heterogeneity—given the tissue-specific expression patterns of miRNAs—we estimated monocyte proportions in all transcriptomic samples and retained only those individuals with >90% monocyte content (n = 504) for developing and evaluating miRNA expression prediction models. The complete methodological details for monocyte proportion estimation were descripted in our previous study [50] and the Supplementary File. To ensure sample independence, pairwise kinship coefficients were estimated for all participants using KING (v2.2) software [51] with the “--related --degree 3” parameters, which corresponds to a kinship coefficient threshold of >0.044 for detecting third-degree or closer relatives. Individuals were grouped into families based on these relationships, and one individual from each family was retained for downstream analyses. This relatedness filtering removed 392 individuals from the CAU group, 354 from the AA group. The same set of unrelated individuals was used consistently across all subsequent analyses, including the training of genetic imputation models, eQTL mapping, and association analyses, thereby ensuring the validity of sample independence assumptions.

The final set of unrelated, high-monocyte individuals was stratified by self-reported ethnicity into two groups: CAU (n = 281) and AA (n = 170). The trained models were then applied to impute miRNA expression levels in the remaining LOS participants who had WGS data but no transcriptomic data, comprising 3487 CAU and 2405 AA individuals for downstream association analyses. For all participants, BMD and lean mass was measured for all participants by trained and certified staff using dual-energy X-ray absorptiometry (DXA; Hologic QDR-4500 Discovery, Hologic Inc., Bedford, MA, USA). All participants signed an informed-consent document before any data collection, and the study was approved by the Tulane University Institutional Review Board and was in accordance with the Declaration of the Helsinki. Demographic and clinical characteristics of the analyzed cohorts are summarized in Table 2.

### 4.2. Genotyping and Quality Control

DNA isolation and sequencing of LOS samples has been described previously [25]. DNA extracted from blood samples was sequenced on a BGISEQ-500 platform (BGI Americas Corporation, Cambridge, MA, USA) at an average read depth of 15×, producing paired-end 350 bp reads across two sequencing runs. Genetic variants were identified in accordance with the Genome Analysis Toolkit versions 4 (GATK4) best practices for variant calling. Variant quality score recalibration (VQSR) was applied to remove potential sequencing artifacts and retain high-confidence variant calls. The sample-level WGS QC procedures were provided in the Supplementary File. Additional quality control was performed using Plink 2.0 based on the following criteria: (1) minor allele frequency > 1%; (2) variant missing rate < 10%; and (3) Hardy–Weinberg equilibrium test *p*-value > 1 × 10^−5^.

### 4.3. Small RNA-Seq Library Construction, Sequencing, and Primary Data Processing

Small RNA sequencing libraries were prepared using TruSeq Small RNA Sample Prep Kits (Illumina, San Diego, CA, USA). The constructed libraries were sequenced using Illumina Hiseq2000/2500 following the manufacturer’s protocol. Raw reads were subjected to an in-house program, ACGT101-miR (LC Sciences, Houston, TX, USA) to remove adapter dimers, junk, low complexity, common RNA families (rRNA, tRNA, snRNA, snoRNA) and repeats. Subsequently, unique sequences with length in 18~26 nucleotide were mapped to specific species precursors in miRBase (v22) [52] by BLAST search to identify known and novel miRNAs. The original copy numbers were corrected followed the procedures as described in a previous study [53] and the Supplementary File.

### 4.4. Quality Control and Normalization of MiRNA Expression Data

Our analysis focused on 3259 miRNAs that were mapped to annotated human miRNAs in reference databases [52]. The following quality control and normalization procedures were applied:

(a) Removal of lowly expressed miRNAs that are lowly expressed in the majority of samples (with corrected copy number counts < 1 in more than 80% of samples);
(b) Exclusion of miRNAs located on sex chromosomes;
(c) Log_2_-transformation of expression counts, followed by estimation of hidden confounders using the probabilistic estimation of expression residuals (PEER) method (45 factors for the CAU cohort, 30 for the AA cohort) [54]. The number of PEER factors was selected based on GTEx Consortium recommendations and validated by examining variance explained curves, which confirmed that the selected factors captured the majority of hidden confounding with minimal additional variance explained beyond these thresholds (Figure S1);
(d) Regression of the log-transformed miRNA expression values against age, the estimated PEER factors, and the top 10 genetic principal components (PCs). The resulting residuals were subsequently rank-based inverse normal transformed (INT) to mitigate the influence of extreme values. Prediction models were trained using both the INT-residuals and the non-transformed residuals; however, all downstream association analyses were performed exclusively using the INT-based models to ensure compliance with the normality assumptions underlying linear regression and to maintain consistency with standard eQTL mapping practices. Consequently, in these INT-based models, the β coefficient for a genetic variant represents the expected change in the residualized miRNA expression, in standard deviation units, per copy of the effect allele.

Following this pipeline, a total of 837 high-quality human miRNAs were retained for subsequent investigation.

### 4.5. Identification of Significant MiRNA-eQTLs

A miRNA was considered significant if it harbored at least one significant cis-miRNA-eQTL within a 1000 kb window upstream or downstream of the pre-miRNA genomic locus. Gene coordinates based on the GRCh38 reference genome were obtained from the Ensembl BioMart database [55]. Association testing was performed using the adaptive permutation procedure implemented in QTLtools v1.2 [56] with an adaptive permutation procedure. Briefly, we performed up to 10,000 permutations per miRNA to empirically establish the null distribution of the strongest association signal. The adaptive algorithm evaluates significance iteratively and may stop early when the *p*-value can be reliably estimated. The permutation results were fitted to a beta distribution, and nominal *p*-values were derived from this fitted distribution, adjusting for the number of variants tested in cis. Finally, genome-wide false discovery rate (FDR) correction was applied using the Benjamini–Hochberg method via the QTLtools. Significant miRNA-eQTLs were defined as those passing a FDR threshold of <5%.

### 4.6. Mapping of Independent Cis-miRNA-eQTL Signals

The significant miRNAs may have multiple proximal cis-SNPs (within ±1000 kb nearby the pre-miRNA) which have independent effects. For miRNAs with significant cis-eQTLs, we further identified independent regulatory signals among the proximal SNPs using the conditional analysis approach in QTLtools. This method employs a forward-backward stepwise regression to select the conditional independent signals using this significance threshold. In this process, it automatically learns the number of independent signals per miRNA using forward selection, and then determines the best candidate SNP per signal using backward selection controlling for the remaining signals. If no SNP is significant at the previous nominal *p*-value threshold, the candidate signal will be dropped; otherwise, the SNP with smallest backward-*p*-value will be chosen as the lead SNP for this candidate signal. In some cases, the same SNP during the backward selection can explain multiple independent signals that were detected during the forward selection.

### 4.7. Estimation of Cis-SNP Heritability and Training of Imputation Models

Cis-SNP heritability (cis-h^2^), representing the proportion of miRNA expression variance explained by cis-SNPs, was estimated using the restricted maximum likelihood (REML) algorithm implemented in GCTA [57]. Subsequently, genetic prediction models for miRNA expression were constructed using the TWAS/FUSION framework [23]. For each miRNA, a model was trained by integrating five complementary approaches: best linear unbiased prediction (BLUP), least absolute shrinkage and selection operator (LASSO), elastic net (ENET), top single nucleotide polymorphism (TOP1), and Bayesian sparse linear mixed model (BSLMM). Since a mature miRNA may originate from multiple precursors, each with distinct regulatory mechanisms, we constructed a separate imputation model for each miRNA precursor (pre-miRNA). The set of predictive features included all cis-SNPs within a 1 Mb window (±1000 kb) of the pre-miRNA. To improve predictive accuracy, we further incorporated trans-SNPs identified from the prior QTL analysis at a suggestive significance threshold (*p* < 1 × 10^−5^). The model with the highest R^2^ in 5-fold cross-validation was selected as the optimal predictor for each miRNA. Finally, we retained models for downstream association analysis only if the model achieved a cross-validation R^2^ ≥ 0.01 and a cross-validated nominal *p* < 0.05—a conventional threshold in transcriptome-wide prediction studies [58].

### 4.8. Association Between Predicted MiRNA Expression and Musculoskeletal Diseases

We first employ the miRNA imputation models in association analyses using individual-level genetic data. The trained miRNA expression prediction models were applied to the remaining LOS participants with only WGS data (3487 CAU and 2405 AA individuals). Next, we evaluated the associations between imputed miRNA expression levels and two musculoskeletal diseases: osteoporosis (FNK-BMD, SPN-BMD, and HIP-BMD), and sarcopenia (ALM and grip strength). Regression model was performed to test the association between predicted miRNA and each trait, corrected with the following covariates: age, height, weight, exercise regular, smoking, alcohol, and the top 10 genetic PCs. Variance inflation factor (VIF) analysis confirmed that all included covariates had VIF values below 5, indicating no significant multicollinearity concerns (Figure S2).

We further integrated the trained miRNA imputation models with the public large-scale public GWAS summary statistics to test the association between miRNA and diseases, using the association test approach derived in FUSION [23]. For osteoporosis, we incorporated FNK- and SPN-BMD summary statistics from our previous study (n = 17,964) [59], TB-BMD data from a life-course meta-analysis (n = 66,628, primarily European) [60], and eBMD data from the GEFOS database (http://www.gefos.org, including 426,824 European-ancestry participants) [61]. For sarcopenia, ALM summary statistics were obtained from the GWAS catalog (GCST90000025, n = 450,243 European-ancestry individuals) [62]. For each GWAS, the LD reference panel was selected to match the ancestry of the miRNA imputation model: the European-ancestry LD reference panel from the LOS training set was applied when using CAU-trained models, while the African-ancestry LD reference panel was applied for AA-trained models. Raw *p*-values from the association analyses were corrected for multiple testing using the Benjamini–Hochberg (BH) method. The miRNAs with an FDR-adjusted *p*-value (FDR) < 0.05 were considered statistically significant.

### 4.9. Identification of Target Genes for MiRNA

To identify target genes of the trait-associated miRNAs, we employed three complementary prediction tools: TargetScan (v8.0) [26], miRDB (v6.0) [27], and miRTarBase (Release 10.0) [28]. For each tool, predicted targets were filtered according to established scoring thresholds to ensure high confidence. Specifically, in TargetScan, only genes with a total context++ score < −0.1 were retained. For miRDB, low-confidence targets (confidence score < 60) were excluded. In miRTarBase, only targets supported by strong experimental evidence were considered. These tools utilize different algorithms and databases to provide a comprehensive analysis of miRNA-target interactions. To optimize the sensitivity of our predictions and minimize potential false negatives, we integrated the results from all three databases, selecting the union of all predicted targets given the minimum overlap. This integrative approach helps in identifying a more reliable set of potential targets by leveraging the strengths and minimizing the weaknesses of each prediction tool.

### 4.10. Association Between Target Genes and Musculoskeletal Diseases

Given that a single disease-associated miRNA may regulate hundreds of potential target genes—not all of which are relevant to the disease—we performed an additional analysis to test the association between the targets and the corresponding traits, using ancestry-stratified transcriptome profiles from the LOS cohort (AA, n = 170; CAU, n = 281).

The mRNA sequencing reads were preprocessed and normalized using edgeR package [63]. Association testing was conducted using linear models with limma package [64], adjusting for a set of covariates: age, height, weight, exercise regular, smoking, alcohol, and the top 10 genetic PCs.

### 4.11. Functional Enrichment of Target Genes

To characterize the biological functions of the target genes, we performed enrichment analysis using the GO database [65], via the Database for Annotation, Visualization and Integrated Discovery (DAVID; https://davidbioinformatics.nih.gov; accessed on 12 March 2026) platform [66]. The GO resource offers a computational framework representing the functional roles of genes in humans and other organisms. The GO resource covers three biological domains: Molecular function (MF), which describes activities that occur at the molecular level; cellular component (CC), which is related to cellular structures in which a gene product performs a function, either cellular compartments, or stable macromolecular complexes of which they are parts; and biological process (BP), which is accomplished by multiple molecular activities. To obtain mechanistically informative terms, we applied the GOTERM_FAT option, which automatically excludes overly broad and non-specific GO categories. To assess the robustness of the enrichment, we used two distinct background gene sets: (i) all human genes (the default setting) and (ii) all of the target genes for the identified diseases-related miRNAs in our analyses. Only GO terms that were significantly enriched (*p* < 0.05) under both background conditions were retained.

### 4.12. Identification of Therapeutic Drugs for MiRNA-Targeted Genes

To evaluate the therapeutic potential of miRNA-targeted genes, we queried the DGIdb (Version 2024-Dec) to identify drugs associated with each candidate gene. The DGIdb streamlines the search for druggable therapeutic targets through the aggregation, categorization, and curation of drug and gene data from publications and expert resources, containing over 10,000 genes and 20,000 drugs involved in over 70,000 drug-gene interactions [37]. Only approved drugs were retained for subsequent analysis.

## 5. Conclusions

Our study demonstrates the value of large and diverse ancestry study for genetic mechanisms of molecular phenotypes and their relationship with complex diseases. We also provide comprehensive and cross-ancestral insights into the genetic architecture of miRNAs in monocytes. The miRNAs and their targets associated with musculoskeletal diseases identified in our study not only expand the repertoire of potential biomarkers for these diseases but also offer new perspectives for identifying therapeutic targets. The generated miRNA-eQTL resources and imputation models serve as a foundational tool for the scientific community for other diseases, enabling future studies to interrogate miRNA-disease relationships and to explore the drug repurposing potential of targeting these miRNA-mediated pathways.

## Figures and Tables

**Table 1:** Significant trait-associated miRNAs.

PANEL	Analysis Method	Trait	Data Source	Sex	Count	Significant Mature miRNAs
CAU	Individual-level predicted miRNA	FNK-BMD	LOS	Female	1	hsa-miR-323a-3p
		HIP-BMD		Female	1	hsa-miR-323a-3p
		FNK-BMD		Male	1	hsa-miR-4701-5p
		SPN-BMD		Male	1	hsa-miR-7976
	Summary-level GWAS statistics	eBMD	GEFOS	Mixed	19	hsa-miR-10399-5p, hsa-miR-1185-1-3p, hsa-miR-1287-5p, hsa-miR-141-3p, hsa-miR-146a-5p, hsa-miR-185-5p, hsa-miR-210-5p, hsa-miR-26b-3p, hsa-miR-31-5p, hsa-miR-3177-3p, hsa-miR-4440, hsa-miR-4665-5p, hsa-miR-4799-5p, hsa-miR-485-5p, hsa-miR-497-5p, hsa-miR-548ae-5p, hsa-mir-641-p3, hsa-miR-6513-5p, hsa-miR-6516-3p
		ALM	GWAScatlog	Mixed	26	hsa-let-7a-3p, hsa-miR-1185-2-3p, hsa-miR-127-5p, hsa-miR-146b-5p, hsa-miR-15a-3p, hsa-miR-17-3p, hsa-miR-19a-3p, hsa-miR-23b-3p, hsa-miR-24-2-5p, hsa-miR-26a-5p, hsa-miR-26b-3p, hsa-miR-27b-3p, hsa-miR-3138, hsa-miR-335-3p, hsa-miR-338-5p, hsa-miR-376b-5p, hsa-miR-376c-5p, hsa-miR-4677-3p, hsa-miR-548au-5p, hsa-miR-548k, hsa-miR-550a-3-5p, hsa-miR-590-5p, hsa-miR-627-5p, hsa-miR-6513-3p, hsa-miR-6513-5p, hsa-miR-744-5p
AA	Individual-level predicted miRNA	SPN-BMD	LOS	Male	1	hsa-miR-335-3p
	Summary-level GWAS statistics	eBMD	GEFOS	Mixed	25	hsa-miR-103a-3p, hsa-miR-1260a, hsa-miR-1268b, hsa-miR-1285-3p, hsa-miR-1296-5p, hsa-miR-148b-3p, hsa-miR-195-5p, hsa-miR-25-5p, hsa-miR-29c-3p, hsa-miR-323a-3p, hsa-miR-338-3p, hsa-miR-342-3p, hsa-miR-3605-3p, hsa-miR-365a-3p, hsa-miR-432-5p, hsa-miR-4448, hsa-miR-4508, hsa-miR-486-3p, hsa-miR-548ae-5p, hsa-miR-589-3p, hsa-miR-6513-3p, hsa-miR-6516-3p, hsa-miR-6813-5p, hsa-miR-7706, hsa-miR-941
		ALM	GWAScatlog	Mixed	14	hsa-miR-1296-5p, hsa-miR-150-3p, hsa-miR-185-5p, hsa-miR-200c-3p, hsa-miR-25-5p, hsa-miR-29c-3p, hsa-miR-3074-5p, hsa-miR-30d-5p, hsa-miR-30e-5p, hsa-miR-425-3p, hsa-miR-589-3p, hsa-miR-6501-5p, hsa-miR-6513-3p, hsa-miR-769-5p

CAU refers to Caucasian; AA refers to African American; BMI refers to body mass index; ALM refers to appendicular lean mass; FNK refers to femoral neck; SPN refers to spine; BMD refers to bone mineral density; eBMD refers to heel estimated BMD.

**Table 2:** Demographic and clinical characteristics of LOS cohort.

Dataset	Model Training		Association Testing			
Race	CAU	AA	CAU		AA	
Sex	Male	Male	Male	Female	Male	Female
Sample size	281	170	1651	1836	1347	1058
Age (year)	35.54 (8.69)	39.48 (7.53)	42.48 (14.37)	45.59 (15.99)	44.66 (11.71)	43.92 (14.19)
Height (cm)	175.73 (7.05)	175.68 (7.12)	175.73 (6.92)	163.15 (6.69)	175.19 (7.25)	163.52 (6.48)
Weight (kg)	82.77 (15.64)	83.38 (16.71)	83.92 (16.46)	69.83 (17.6)	84.22 (19.88)	84.03 (21.85)
BMI (kg/m^2^)	26.82 (5.07)	27.02 (5.2)	27.15 (4.95)	26.24 (6.49)	27.38 (5.94)	31.42 (7.94)
ALM (kg)	27.74 (4.22)	30.27 (4.63)	27.7 (4.46)	18.81 (3.6)	29.61 (5.41)	22.6 (4.86)
Grip strength (kg)	39.96 (9.14)	39.75 (10.26)	41.33 (12.05)	26.59 (8.17)	40.86 (13.16)	26.48 (9.54)
Regular Exercise (%)	224 (79.72)	114 (67.06)	1240 (75.11)	1401 (76.31)	955 (70.9)	638 (60.3)
Smoking (%)	191 (67.97)	135 (79.41)	1079 (65.35)	727 (39.6)	994 (73.79)	371 (35.07)
Alcohol drinking (%)	203 (72.24)	92 (54.12)	1300 (78.74)	1572 (85.62)	862 (63.99)	592 (55.95)
FNK-BMD (g/cm^2^)	0.85 (0.13)	0.94 (0.14)	0.85 (0.2)	0.78 (0.19)	0.94 (0.23)	0.88 (0.15)
HIP-BMD (g/cm^2^)	0.98 (0.12)	1.06 (0.13)	1 (0.15)	0.91 (0.19)	1.07 (0.17)	1.01 (0.16)
SPN-BMD (g/cm^2^)	1.01 (0.14)	1.07 (0.12)	1.05 (0.15)	1 (0.17)	1.11 (0.17)	1.08 (0.17)

Data are presented as mean (Standard deviation, SD) for continuous variables and n (%) for categorical variables. CAU refers to Caucasian; AA refers to African American; BMI refers to body mass index; ALM refers to appendicular lean mass; FNK refers to femoral neck; SPN refers to spine; BMD refers to bone mineral density.

## Data Availability

The datasets from LOS that support this study’s findings are available from the principal investigator (H.W.D., hdeng2@tulane.edu) upon reasonable request. Access will be granted for academic research purposes, subject to IRB approval and the completion of a data use agreement. Additionally, the LOS WGS data are in the process of being deposited in the AgingResearchBiobank (https://agingresearchbiobank.nia.nih.gov/), where it will be made available to qualified researchers upon application and approval. The eQTL results and trained miRNA imputation models are publicly available in Mendeley Data at https://data.mendeley.com/datasets/2zpdtdtjwh/3 (accessed on 14 March 2026).

## Supplementary Materials

The following supporting information can be downloaded at: https://www.mdpi.com/article/10.3390/ijms27062818/s1. References [67,68,69,70,71,72,73] are cited in the supplementary materials.

## Abbreviations

The following abbreviations are used in this manuscript:

GWAS	Genome-wide association study
AA	African American
ALM	Appendicular lean mass
BH	Benjamini–Hochberg
BLUP	Best linear unbiased prediction
BMD	Bone mineral density
BMI	Body mass index
BP	Biological process
BSLMM	Bayesian sparse linear mixed model
CAU	Caucasian
CC	Cellular component
DGIdb	Drug-Gene Interaction Database
eBMD	BMD estimated from quantitative heel ultrasounds
ENET	Elastic net
eQTLs	expression quantitative trait loci
FDR	False discovery rate
FNK-BMD	BMD at femoral neck
GATK	Genome Analysis Toolkit
GO	Gene Ontology
h^2^	Heritability
HIP-BMD	BMD at hip
LASSO	Least absolute shrinkage and selection operator
LD	Linkage disequilibrium
LOS	Louisiana Osteoporosis Study
MF	Molecular function
MiRNAs	MicroRNAs
PC	Principal component
PEER	Probabilistic estimation of expression residual
Pre-miRNA	MiRNA precursor
QKI	Quaking
RANKL	NF-κB ligand
REML	Restricted maximum likelihood
SNP	single nucleotide polymorphism
SPN-BMD	BMD at spine
TB-BMD	Total body BMD
TOP1	Top single nucleotide polymorphism
TWAS	Transcriptome-wide association study
UKBB	UK Biobank
VIF	Variance inflation factor
VQSR	Variant quality score recalibration
WGS	Whole-genome sequencing
WTS	Whole-transcriptome sequencing
