# Supplementary material for: Genetic Regulation of Monocyte MicroRNAs and Their Implication in Musculoskeletal Diseases: A Cross-Ancestry Expression Quantitative Trait Loci and Imputation Study
Source: Int J Mol Sci. 2026 Mar 20;27(6):2818. doi: 10.3390/ijms27062818 (PMC13026450; doi:10.3390/ijms27062818)
Supplement: Supplementary file 1 [file ijms-27-02818-s001.zip › Supplementary File.pdf]

International Journal of *Molecular Sciences*

MDPI

Supplementary Materials

Genetic Regulation of Monocyte microRNAs and Their Implication in Musculoskeletal Diseases: A Cross-Ancestry Expression Quantitative Trait Loci and Imputation Study

Yong Liu [1], Kuan-Jui Su [2], Yun Gong [2], Bo Tian [1], Anqi Liu [2], Zhe Luo [2], Qing Tian [2], Chuan Qiu [2], Hui Shen [2], Hong-Mei Xiao [3] and Hong-Wen Deng [2]*

[1] Center for System Biology, Data Sciences, and Reproductive Health, School of Basic Medical Science, Central South University, Changsha 410031, China.
[2] Tulane Center of Biomedical Informatics and Genomics, Deming Department of Medicine, School of Medicine, Tulane University, New Orleans, LA 70112, USA.
[3] Institute of Reproductive & Stem Cell Engineering, school of basic medical science, Central South University, Changsha, 410000, China.

* Correspondence: hdeng2@tulane.edu.

1. Supplementary Material and Methods

1.1. Isolation of Monocytes, Their Genomic DNA, and Total RNA

This study focused on peripheral blood monocytes (PBMs), which can serve as osteoclast precursors and play important roles in regulating bone metabolism [66,67]. Briefly, peripheral blood mononuclear cells (PBMCs) were first isolated from approximately 60 mL of freshly collected peripheral blood using density gradient centrifugation with Histopaque-1077 (Sigma-Aldrich, USA). The PBMCs were washed repeatedly with PBS containing 2 mM EDTA, then resuspended in PBS supplemented with 0.5% BSA and 2 mM EDTA. PBMs were subsequently isolated from the PBMCs using a Monocyte Isolation Kit II (Miltenyi Biotec GmbH, Bergisch Gladbach, Germany) according to the manufacturer's instructions. This kit depletes unwanted cells (e.g., T and B cells) from PBMCs, yielding PBMs free of surface-bound antibodies and beads with minimal disturbance. The purity of the isolated PBMs was visually assessed and counted under a microscope. Genomic DNA for whole-genome bisulfite sequencing (WGBS) and total RNA for RNA sequencing were extracted from freshly isolated PBMs using the AllPrep DNA/RNA/miRNA Universal Kit (Qiagen, USA) following the manufacturer's protocol. All samples were stored at −80 °C until further use.

1.2. Cell deconvolution analysis

Cellular composition was inferred using Bayesian cell proportion reconstruction via statistical marginalization (BayesPrism) [68]. Publicly available single-cell RNA sequencing datasets—PBMCs from Healthy Human, Single Cell Gene Expression Profiling Dataset by Cell Ranger v1.1.0 (10x Genomics, 2016) and PBMCs and Neutrophils, 5' from Healthy Human, Single Cell Immune Profiling Dataset by Cell Ranger v6.1.0 (10x Genomics, 2021)—were used as references. The reference datasets were clustered using Seurat v5 [69] and visualized with uniform manifold approximation and projection (UMAP).

Quality control filtering removed cells with fewer than 200 genes, more than 10,000 counts, or greater than 10% mitochondrial content. Data normalization, scaling, and selection of highly variable genes (HVGs) were performed using the SCTransform function in Seurat v5 [69]. The top 3,000 HVGs were selected for downstream analysis, including principal component analysis (PCA). The first 30 principal components were used for dimensionality reduction via UMAP. The two reference datasets were integrated using anchor-based reciprocal PCA (RPCA). Clustering analysis identified 11 clusters, which were annotated into six major cell types: T cells (CD3), B cells (MS4A1), natural killer cells (GNLY), dendritic cells (CD1C, FCER1A, LILRA4), platelets/megakaryocytes (PPBP), CD16+ monocytes (CD16, MS4A7), and CD14+ monocytes (CD14, LYZ). BayesPrism utilizes cell type-specific expression profiles from the scRNA-seq reference to estimate the cellular composition of bulk RNA-seq samples. Subsequently, gene expression profiles were adjusted for CD16+ and CD14+ monocyte populations.

1.3. Sample-level WGS QC procedures

The WGS was conducted at an average read depth of 15× using the DNBSEQ-500 sequencing technology platform (BGI Americas Corporation, Cambridge, MA, USA), with 350 bp paired-end reads. Cleaned reads from each sample were aligned to the human reference genome (GRCh38/hg38) using the Burrows-Wheeler Aligner (BWA, v0.7.12) [70]. For accurate variant calling, we followed the Genome Analysis Toolkit (GATK, v4.0.3) Best Practices [71,72]. HaplotypeCaller was employed to identify genomic variations, and variant quality score recalibration (VQSR) was applied to obtain high-confidence variant calls [71,72]. SNPs and InDels marked as PASS in the output VCF file were retained as high-confidence variant sets. For SNP recalibration, we used the following training datasets and features: (a) Training sets: HapMap V3.3, Omni2.5M genotyping array data, and high-confidence SNP sites from the 1000 Genomes Project; (b) Features: coverage (DP), quality by depth (QD), Fisher strand bias (FS), strand odds ratio (SOR), and read position rank sum test (ReadPosRankSum). Samples that passed the facility's quality thresholds were retained: Q20 and Q30 scores (with ≥80% of bases achieving ≥Q30), GC content (~40%), mapping rate (>99%), duplicate rate (<20%), mismatch rate (<0.5%), average sequencing depth (>15×), genome coverage (>95%), and coverage at least 4×. Samples failing to meet these thresholds were flagged for resequencing or further evaluation.

1.4. Primary data processing for copy numbers of small RNA sequencing reads

The original copy numbers were corrected using the following procedure:

(a) Find a common set of sequences among all samples;
(b) Construct a reference data set. Each data in the reference set is the copy number median value of a corresponding common sequence of all samples;
(c) Perform 2-based logarithm transformation on copy numbers ($\log_2(\text{copy\#})$) of all samples and reference data set;
(d) Calculate the $\log_2(\text{copy\#})$ difference ($|\Delta\log_2(\text{copy\#})|$) between individual sample and the reference data set;
(e) Form a subset of sequences by selecting $|\Delta\log_2(\text{copy\#})| < 2$, which means less than ($2^2 =$) 4-fold change from the reference set;
(f) Perform linear regressions between individual samples and the reference set on the subset sequences to derive linear equations $\mathrm{y} = \mathrm{a}_i\mathrm{x} + \mathrm{b}_i$, where a_i and b_i are the slop and interception, respectively, of the derived line, x is $\log_2(\text{copy\#})$ of the reference set, and y is the expected $\log_2(\text{copy\#})$ of sample i on a corresponding sequence;

https://doi.org/10.3390/ijms27062818

(g) Calculate the mid value $x_{mid} = (\max(x) - \min(x))/2$ of the reference set. Calculate the corresponding expected $\log_2(\text{copy\#})$ of sample i, $y_{i,mid} = a_i x_{mid} + b_i$. Let $y_{r,mid} = x_{mid}$, let $\Delta y_i = y_{r,mid} - y_{i,mid}$, which is the logarithmic correction factor of sample i. We then derive the arithmetic correction factor $f_i = 2^{\Delta y_i}$ of sample i;

(h) Correct copy numbers of individual samples by multiplying corresponding arithmetic correction factor f_i to original copy numbers.

2. Supplementary Figures

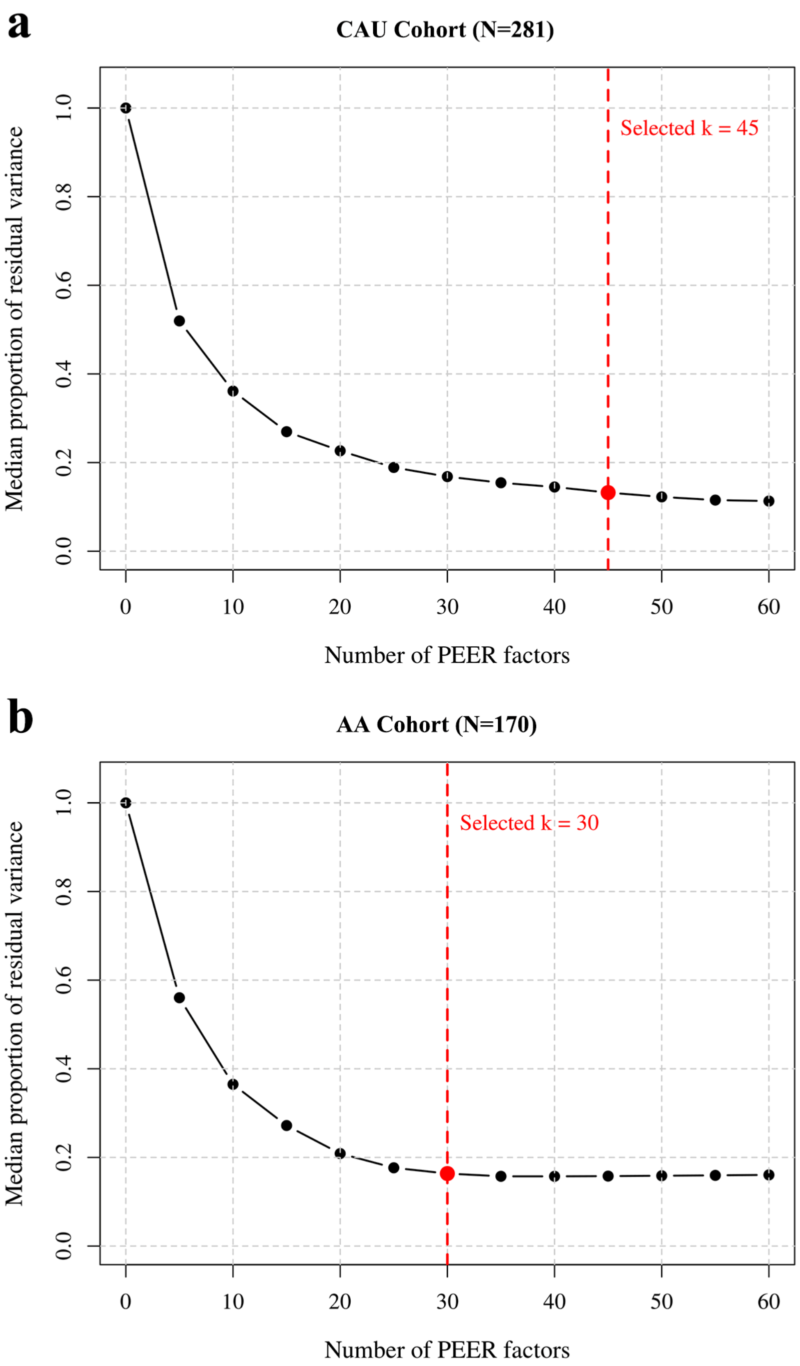

Figure S1. The variance explained curves for PEER analysis in (a) CAU and (b) AA populations. The vertical dashed lines indicate the selected numbers of PEER factors (45 for

CAU, 30 for AA). Beyond these thresholds, the variance explained plateaus, suggesting that additional factors provide minimal benefit and may lead to overfitting.

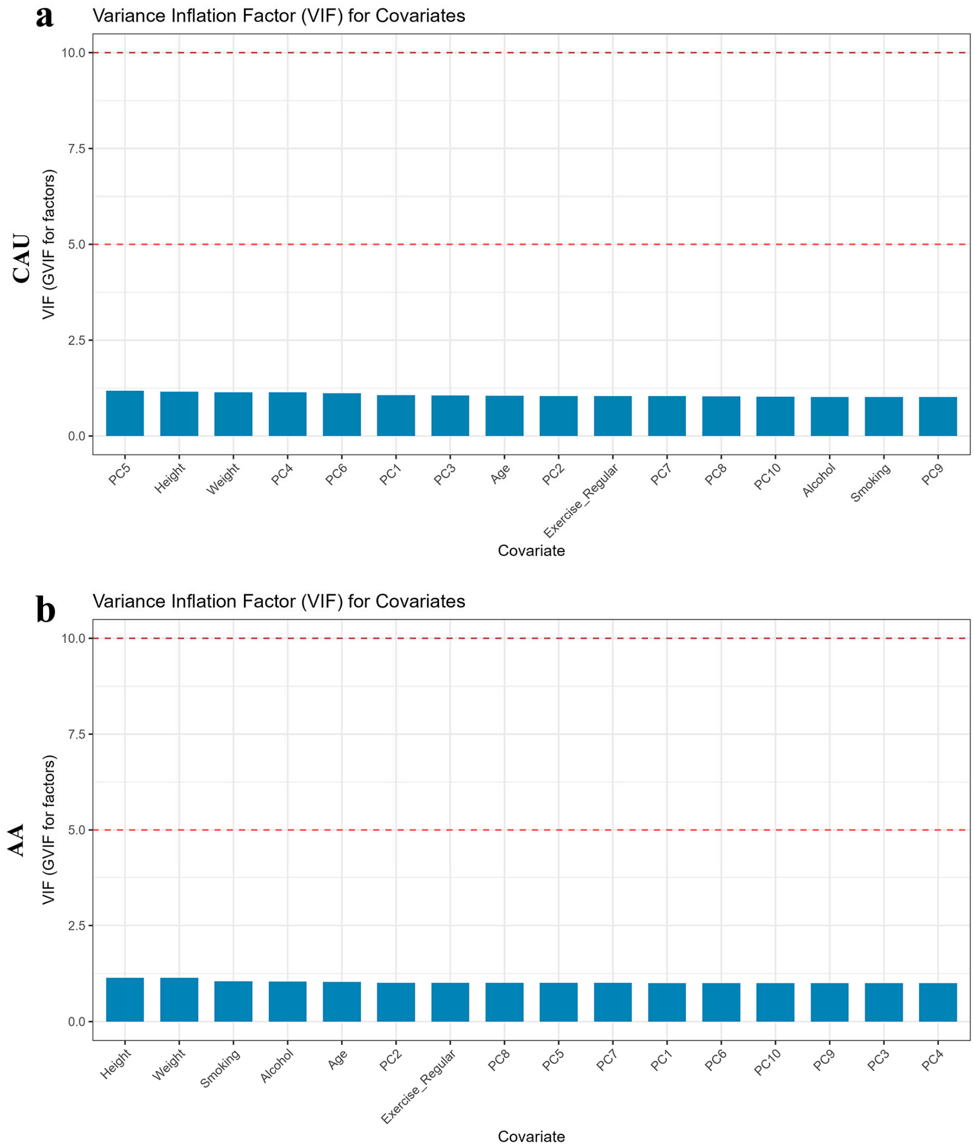

Figure S2. Variance inflation factor (VIF) for covariates in (a) CAU and (b) AA populations. Covariates are ordered by descending VIF values. For factor variables (Exercise_Regular, Smoking, Alcohol), the generalized VIF (GVIF) is displayed; for continuous variables, the standard VIF is shown. The red and dark red dashed lines indicate VIF = 5 and VIF = 10, respectively, commonly used thresholds for moderate and high multicollinearity.

https://doi.org/10.3390/ijms27062818

References

67. Fujikawa, Y.; Sabokbar, A.; Neale, S.; Athanasou, N.A. Human Osteoclast Formation and Bone Resorption by Monocytes and Synovial Macrophages in Rheumatoid Arthritis. Ann. Rheum. Dis. 1996, *55*, 816–822, doi:10.1136/ard.55.11.816.

68. Lari, R.; Kitchener, P.D.; Hamilton, J.A. The Proliferative Human Monocyte Subpopulation Contains Osteoclast Precursors. Arthritis Res. Ther. 2009, *11*, doi:10.1186/ar2616.

69. Chu, T.; Wang, Z.; Pe'er, D.; Danko, C.G. Cell Type and Gene Expression Deconvolution with BayesPrism Enables Bayesian Integrative Analysis across Bulk and Single-Cell RNA Sequencing in Oncology. Nat. Cancer 2022, *3*, 505–517, doi:10.1038/s43018-022-00356-3.

70. Hao, Y.; Stuart, T.; Kowalski, M.H.; Choudhary, S.; Hoffman, P.; Hartman, A.; Srivastava, A.; Molla, G.; Madad, S.; Fernandez-Granda, C.; et al. Dictionary Learning for Integrative, Multimodal and Scalable Single-Cell Analysis. Nat. Biotechnol. 2024, *42*, 293–304, doi:10.1038/s41587-023-01767-y.

71. Li, H.; Durbin, R. Fast and Accurate Short Read Alignment with Burrows-Wheeler Transform. Bioinformatics 2009, *25*, 1754–1760, doi:10.1093/bioinformatics/btp324.

72. McKenna, A.; Hanna, M.; Banks, E.; Sivachenko, A.; Cibulskis, K.; Kernytsky, A.; Garimella, K.; Altshuler, D.; Gabriel, S.; Daly, M.; et al. The Genome Analysis Toolkit: A MapReduce Framework for Analyzing next-Generation DNA Sequencing Data. Genome Res. 2010, *20*, 1297–1303, doi:10.1101/gr.107524.110.

73. Van der Auwera, G.A.; Carneiro, M.O.; Hartl, C.; Poplin, R.; del Angel, G.; Levy-Moonshine, A.; Jordan, T.; Shakir, K.; Roazen, D.; Thibault, J.; et al. From FastQ Data to High-Confidence Variant Calls: The Genome Analysis Toolkit Best Practices Pipeline. Curr. Protoc. Bioinforma. 2013, *43*, 11.10.1-11.10.33, doi:10.1002/0471250953.bi1110s43.

https://doi.org/10.3390/ijms27062818
